# Structure, Topology, and Dynamics of Membrane-Inserted Polypeptides and Lipids by Solid-State NMR Spectroscopy: Investigations of the Transmembrane Domains of the DQ Beta-1 Subunit of the MHC II Receptor and of the COP I Protein p24

**DOI:** 10.3389/fmolb.2019.00083

**Published:** 2019-09-24

**Authors:** Evgeniy S. Salnikov, Christopher Aisenbrey, Bianca Pokrandt, Britta Brügger, Burkhard Bechinger

**Affiliations:** ^1^Université de Strasbourg/CNRS, UMR7177, Institut de Chimie, Strasbourg, France; ^2^Biochemiezentrum der Universität Heidelberg, Heidelberg, Germany

**Keywords:** transmembrane dimer, highly specific protein-lipid interaction, sphingomyelin recognition motif, supported lipid bilayer, solid-state NMR, helix topology, fatty acyl chain order parameter

## Abstract

MHC class II receptors carry important function in adaptive immunity and their malfunctioning is associated with diabetes type I, chronic inflammatory diseases and other autoimmune diseases. The protein assembles from the DQ alpha-1 and DQ beta-1 subunits where the transmembrane domains of these type I membrane proteins have been shown to be involved in homo- and heterodimer formation. Furthermore, the DQ alpha 1 chain carries a sequence motif that has been first identified in the context of p24, a protein involved in the formation of COPI vesicles of the intracellular transport machinery, to specifically interact with sphingomyelin-C18 (SM-C18). Here we investigated the membrane interactions and dynamics of DQ beta-1 in liquid crystalline POPC phospholipid bilayers by oriented ^15^N solid-state NMR spectroscopy. The ^15^N resonances are indicative of a helical tilt angle of the membrane anchor sequence around 20°. Two populations can be distinguished by their differential dynamics probably corresponding the DQ beta-1 mono- and homodimer. Whereas, this equilibrium is hardly affected by the addition of 5 mole% SM-C18 a single population is visible in DMPC lipid bilayers suggesting that the lipid saturation is an important parameter. Furthermore, the DQ alpha-1, DQ beta-1 and p24 transmembrane helical domains were reconstituted into POPC or POPC/SM-C18 lipid bilayers where the fatty acyl chain of either the phosphatidylcholine or of the sphingolipid have been deuterated. Interestingly in the presence of both sphingolipid and polypeptide a strong decrease in the innermost membrane order of the POPC palmitoyl chain is observed, an effect that is strongest for DQ beta-1. In contrast, for the first time the polypeptide interactions were monitored by deuteration of the stearoyl chain of SM-C18. The resulting ^2^H solid-state NMR spectra show an increase in order for p24 and DQ alpha-1 which both carry the SM recognition motif. Thereby the data are suggestive that SM-C18 together with the transmembrane domains form structures imposing positive curvature strain on the surrounding POPC lipids. This effect is attenuated when SM-C18 is recognized by the protein.

## Introduction

Membrane protein function is not only dependent on the global and local phase properties of the membrane but also on the immediate lipid environment (Lingwood and Simons, 2010; Iversen et al., 2015), where the activity of proteins proteins can be modulated by specific lipid interactions (Hunte and Richers, 2008; Ekanayake et al., 2016; Jaipuria et al., 2017). The residence time of lipids in the proteins proximity determines if it is considered a bulk lipid. Alternatively, specific interactions of the protein with polar head groups of lipids and/or by hydrophobic matching to their hydrocarbon chains become important (Jensen and Mouritsen, 2004). Individual lipid molecules have been detected in crystallographic structures. These are located at the periphery or the oligomerisation interface of membrane proteins (Palsdottir et al., 2003). Indeed, activities of a number of channels or enzymes have been found to depend on specific lipid interactions (for reviews see Hille et al., 2015; Stangl and Schneider, 2015).

A highly selective lipid-binding motif recognizing both the head group and the exact length of the fatty acyl chain (18 C atoms) of N-stearoyl-sphingomyelin (SM-C18) has been identified within p24, a member of the p24 family (Contreras et al., 2012). These type-I membrane proteins are associated with coat protein (COP) I transport vesicles where within the early secretory pathway they shuttle between the early Golgi and the endoplasmatic reticulum (Strating and Martens, 2009). Recognition of SM-C18 is assured by the carboxy-terminus of the transmembrane region of p24 where the amino acid motif VXXTLXXIY has been identified to be involved in lipid binding (Contreras et al., 2012).

In follow-up studies a number of protein candidates with signatures homologous to this highly specific sphingolipid-recognition motif were identified by a bioinformatics approach (Bjorkholm et al., 2014). The majority of these proteins are associated with organelles of the secretory pathway and the plasma membrane, including membrane anchor domains of the major histocompatibility complex class II (MHC II). The transmembrane domain of the MHC class II DQ alpha 1 chain (DQA1) assembles with the DQ beta 1 chain (DQB1) as a heterodimer through GXXXG-mediated protein-protein interactions (Russ and Engelman, 2000; Dixon and Roy, 2019) where DQA1 carries the protein's sphingomyelin-C18 interaction motif (Bjorkholm et al., 2014). A cholesterol-recognition motif has also been identified within the C-terminal regions of the TMDs of DQA1 and DQB1 (Roy et al., 2013, 2016).

The MHC class II molecules play an important role in the adaptive immune response. As a consequence, the malfunctioning of these receptors is associated with diabetes type I, chronic inflammatory and other autoimmune diseases (King and Dixon, 2010; Tsai and Santamaria, 2013). An increased risk for type I diabetes is indeed also associated with single amino acid alterations localized within the putative sphingolipid-binding motif of the transmembrane domain of DQA1 (e.g., Gambelunghe et al., 2007; Diaz-Horta et al., 2010; Dixon et al., 2014).

A better understanding of the structure, lipid interactions and regulation of membrane proteins with important biomedical functions such as MHC class II receptors promises novel insight into the regulatory processes that rule its biological activities and could potentially be used for therapeutic intervention. Whereas, for MHC II the immunogenic peptide binding to its extracellular domains has been investigated in great detail (Painter and Stern, 2012), less is known about the transmembrane domains and their interactions with lipids during assembly, trafficking, peptide loading and presentation (Anderson and Roche, 2015; Dixon and Roy, 2019). The assembly of transmembrane domains into heterodimers, the presence of distinct conformational subclasses and their correlation with biological function have been studied by biophysical approaches, site directed mutagenesis and the analysis of epitope expression (Drake and Drake, 2016) while little is known about the role of sphingomyelin in such process and how SM-C18 interacts with the transmembrane domains.

Recently we have started to investigate the secondary structure, topology and oligomerization of the MHC class II transmembrane domains by CD- and solid-state NMR spectroscopies (Aisenbrey et al., 2019c; Salnikov et al., 2019). In a previous publication two concentration-dependent membrane topologies of the DQA1 transmembrane helical sequence could be identified, suggesting that the transmembrane domain forms homodimers through its GXXXG motif (Travers et al., 1984; King and Dixon, 2010; Dixon et al., 2014; Drake and Drake, 2016; Aisenbrey et al., 2019c; Dixon and Roy, 2019). Here, focus is on DQB1, the second TMD of the MHC II receptor, whose structure and topology was investigated by solid-state NMR spectroscopy on uniaxially aligned samples, a technique that has already revealed the existence of conformational/topological equilibria of DQA1 (Aisenbrey et al., 2019c). The technique has been developed to measure angular restraints that allow for a detailed topological and structural analysis of membrane polypeptides (Aisenbrey et al., 2006; Ramamoorthy et al., 2010; Michalek et al., 2013; Das et al., 2015; Gopinath et al., 2015). In the present work particular emphasis is given to sample heterogeneity and motions. These investigations reveal that the topological equilibria of the transmembrane helix of DQB1 have a different flavor but are as complex as those found for the corresponding DQA1 domain.

Importantly, in this paper for the first time the specific interactions of the transmembrane domains of p24, DQA1, and DQB1 have been investigated in detail by ^2^H solid-state NMR of SM-C18 deuterated at the stearoyl chain. The order parameter profiles that can be obtained by ^2^H solid-state NMR spectroscopy provide atomistic insight into the interactions of the protein with lipids (Dufourc, 2008; Salnikov et al., 2009b; Aisenbrey et al., 2019a,c). By comparing the changes in deuterium order parameters of ${\;}^{2}{\text{H}}_{35}$-SM with those of ${\;}^{2}{\text{H}}_{31}$-POPC carrying deuterons at its stearoyl or palmitoyl side chains, respectively (Aisenbrey et al., 2019c) reveals highly specific effects of the peptides in mixtures of two lipids. The data thereby provide important detail on the structure and dynamics not only of the polypeptides but also of the bilayer lipid components. To our knowledge, the comparison of the POPC and SM-C18 interactions with DQA1, DQB1, and p24 reveal for the first time interesting details related to the specific SM-C18 recognition motif. By investigating the structure, topology and dynamics of both the transmembrane sequence and its surrounding lipids a more comprehensive picture of the regulatory interactions emerges.

## Materials and Methods

### Peptides and Lipids

Natural abundance and palmitoyl chain-deuterated 1-palmitoyl-2-oleoyl-*sn*-glycero-3-phosphocholine (POPC), 1,2-dimyristoyl- *sn*-glycero-3-phosphocholine (DMPC) and ^2^H-depleted water (<1 ppm) were from Sigma-Aldrich (St. Quentin Fallavier France), natural abundance N-octadecanoyl-D-erythro-sphingosylphosphorylcholine (SM-C18) was from Avanti Polar Lipids (Birmingham, AL). The preparation of deuterated SM-C18 is described below. F-moc amino acids were from NovaBiochem/ Merck KGaA (Darmstadt, Germany), isotope labeled amino acids from Cortecnet (Voisins les Bretonneux, France) or Aldrich (Saint Louis, MI, USA), the TentaGel-S-RAM resin used during peptide synthesis from Rapp Polymer GmbH (Tübingen, Germany).

The peptide sequences discussed in this paper are amino acids 215-244 of UniProt entry P01909 (including two additional lysines at both termini, which have been separated from the wt. sequence by a space):

DQA1-TMD: KK TETVV CALGL SVGLV GIVVG TVFII RGLRS KK-CONH_2_.

Amino acids 228-257 of UniProt entry P01920 (including two additional lysines at the N-terminus and one lysine at the C-terminus; the labeled Val and Leu are shown in bold):

DQB1-TMD: KK QSKML SGIGG F**VL**GL IFLGL GLIIH HRSQK K-CONH_2_.

Amino acids 165-193 of UniProt entry Q15363 (including two additional lysines at the amino-terminus):

p24-TMD: KK TNSRV VLWSF FEALV LVAMT LGQIY YLKR-CONH_2._

The predicted transmembrane domains of the three proteins are underlined. Additional lysines at the termini help to solubilize and to handle the peptides.

The sequences were prepared by solid-phase peptide synthesis using a Millipore 9050 automatic peptide synthesizer and a four-fold excess of Fmoc protected amino acid as described previously (Aisenbrey et al., 2019a). To label selected positions with stable isotopes for NMR investigations commercially available Fmoc-protected amino acid precursors carrying ^15^N and/or ^2^H were used at the corresponding coupling steps [Cortecnet (Voisins les Bretonneux, France) or Aldrich (Saint Louis, MI, USA)]. The peptides were purified by reversed phase HPLC (Gilson, Villiers-le-Bel, France) using an acetonitrile/water gradient and a preparative C-18 (Luna, C18-300Å-5 μm, Phenomenex, Le Pecq, France) or a semipreparative C-4 column (Nucleosil C4-300Å-7 μm, Macherey-Nagel, Düren, Germany). The identity and purity of the products [>90% for DQB1; cf. also (Aisenbrey et al., 2019a,c)] were analyzed by HPLC and MALDI mass spectrometry (MALDI-TOF Autoflex, Bruker Daltonics, Bremen, Germany). Before usage the dried peptides were re-dissolved in 4% acetic acid and thoroughly dried to exchange the trifluoroacetic acid counter ions.

### Synthesis of ^2^H_35_-SM-C18

**Scheme 1:**
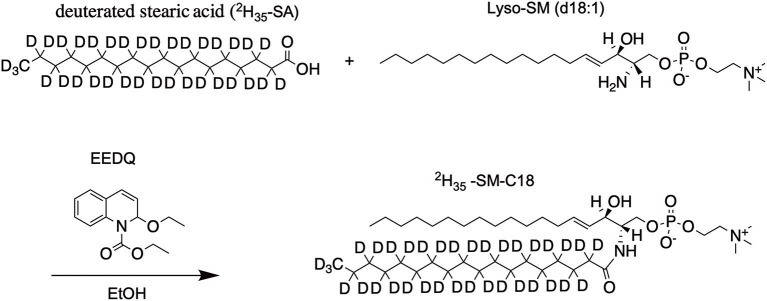
Synthesis and structure of ^2^H_35_-SM-C18.

Lyso-SM (23.5 mg, 50.6 μmol, 1.0 eq) and deuterated stearic acid (33.0 mg, 101.2 μmol, 2.0 eq) were dissolved in 20 mL dry EtOH under an argon atmosphere. 62.5 mg (252.9 μmol, 5.0 eq) of 2-ethoxy-1-ethoxycarbonyl-1,2-dihydroquinoline (EEDQ) was added and after the reaction was stirred 2 h at 45°C an additional portion of EEDQ (62.5 mg, 252.9 μmol, 5.0 eq) was added (Scheme 1). After two more hours at 45°C the reaction mixture was stirred overnight at room temperature. Purification by column chromatography (silica gel 60, 2.5 × 45 cm, DCM/MeOH/H_2_O 50/25/6, visualized by staining with molybdophosphoric acid in 10% EtOH, R_f_ = 0.65) gives the product as a colorless solid (phosphate determination Rouser et al., 1970: 29.9 mg, 39.0 μmol, 77%). ^1^H NMR (600.13 MHz, CDCl_3_): δ = 0.88 (t, *J* = 6.9 Hz, 3H), 1.24–1.30 (m, 22H), 1.92–1.99 (m, 2H), 3.36 (s, 9H), 3.86–4.20 (m, 6H), 4.33–4.46 (m, 2H), 5.39–5.47 (m, 1H), 5.66–5.71 (m, 1H), 7.28–7.50 (bs, 1H) ppm. MS (ESI^+^): m/z C_41_H_48_D_35_N_2_O_6_P + H^+^: calc. 766.83, found 766.84.

### Preparation of Non-oriented Samples

Preparation of non-oriented samples started by mixing 28.5 mg of POPC, 1.5 mg of deuterated SM-C18 (SMd_35_-C18, 5 mol%) and the appropriate amount of peptide (1.45 mg of DQA1 to give 1 mol%; pH adjusted to 7) in hexafluoroisopropanol (HFIP). The solvent was evaporated by exposure to a stream of nitrogen and to high vacuum overnight in such a manner to form a film along the walls of a glass tube. The sample was resuspended in 142.5 μl of 10 mM phosphate buffer (pH 7.1) and involved vortexing and bath sonication, as well as 5 chill/heat cycles at 0 and 40°C to produce multilamellar vesicles. The glass tube (6 mm outer diameter) with the sample was inserted into the solenoidal coil of the solid-state NMR probe.

### Preparation of Samples for Oriented Solid-State NMR Spectroscopy

DQB1 was reconstituted into membranes by dissolving the appropriate amount of peptide in HFIP/water 1/1 (v/v) which was added in a stepwise manner to a solution of 20-40 mg lipid (POPC, DMPC, or POPC/SM-C18) in HFIP. After each step the sample was vortexed and the solvent partially evaporated following a previously elaborated protocol (Kemayo Koumkoua et al., 2014). After repeating this step four times the viscous sample was spread onto ultrathin cover glasses (8 × 12 mm, thickness 00; Marienfeld, Lauda-Königshofen, Germany), dried by exposure to air and thereafter in high vacuum overnight. The lipid bilayers were equilibrated in an atmosphere of 93% relative humidity of ^2^H-depleted water. In order to achieve hydration of DMPC lipid bilayers the samples were equilibrated at 37°C in order to be in the membrane fluid-phase.

### Solid-State NMR Spectroscopy

Proton-decoupled ^31^P solid-state NMR spectra were acquired at 121.577 MHz using a Bruker Avance wide-bore 300 solid-state NMR spectrometer equipped with a commercial double-resonance flat-coil probe (Bruker, Rheinstetten, Germany) (Bechinger and Opella, 1991). A Hahn-echo pulse sequence (Rance and Byrd, 1983) was used with a π/2 pulse of 5 μs, a spectral width of 100 kHz, an echo delay of 40 μs, an acquisition time of 10.2 ms, and a recycle delay of 3 s. External 85% H_3_PO_4_ at 0 ppm was used for calibration. The temperature was set to 23°C for POPC and POPC/SM-C18, and to 37°C for DMPC samples.

^2^H solid-state NMR spectra of deuterated SM-C18 were acquired using a quadrupole pulse-echo sequence (Davis et al., 1976) with a recycle delay of 0.3 s, an echo time of 100 μs, a dwell time of 0.5 μs and a π/2 pulse of 6.1 μs. Before Fourier transformation of the free induction decay an exponential apodization with line broadening of 100 Hz was applied. The temperature was 37°C.

**Figure 1:**
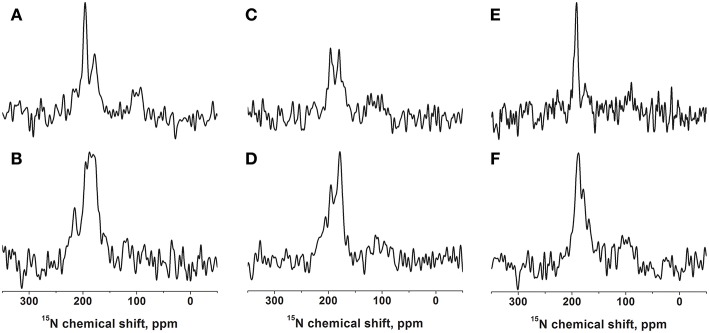
Proton-decoupled cross-polarization ^15^N solid-state NMR spectra of [^15^N-Leu15]-DQB1 reconstituted into 40 mg of phospholipids oriented on glass plates. The membranes are made of POPC **(A,B)**, POPC/SM-C18 95/5 mole/mole **(C,D)**, or DMPC **(E,F)**. The peptide-to-lipid ratio is 1 mole% in the top row **(A,C,E)** and 2 mole% in the second row **(B,D,F)**. Reconstitution followed the protocol described in Kemayo Koumkoua et al. (2014). The membrane normal is aligned parallel to the magnetic field direction. The measurements were performed at room temperature **(A–D)** or at 310 K for the DMPC membranes **(E,F)**.

**Figure 2:**
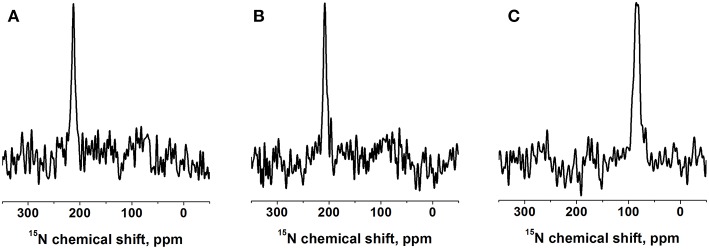
Proton-decoupled ^15^N solid-state NMR spectra of 1 mol% [^15^N-V14]-DQB1 peptide in **(A)** POPC/SM-C18 95/5 mole/mole at 293K, **(B)** DMPC at 310K, both samples were oriented on glass plates and inserted with the membrane normal parallel to the magnetic field direction (B_o_). **(C)** For comparison a bicelle sample made of 2 mol% [^15^N-V14]-DQB1 in DMPC/DHPC at 303K is shown, where the bilayer normal magnetically orients perpendicular to B_o_ (q = 3.2, 28% w/v).

Proton-decoupled ^15^N cross-polarization (CP) spectra were recorded at 76.016 MHz on a Bruker Avance wide bore NMR spectrometer using a cross polarization pulse sequence and an e-free double-resonance flat-coil probe (Gor'kov et al., 2007). The spectral width, acquisition time, CP contact time, and recycle delay time were 100 kHz, 3.9 ms, 0.4 ms, and 2 s, respectively. A field strength of 35 kHz was used during CP, for the ^1^H π/2 pulse and the SPINAL-64 heteronuclear decoupling (Fung et al., 2000). A Lorentz apodization of 200 Hz was applied before Fourier transform. For the acquisition of ^15^N spectra shown in the Figures 1C,F, 2 the acquisition time was extended to 5.2 ms, processing involved a Lorentz apodization of 100 Hz. An external reference of ^15^NH_4_Cl was used for calibration of the ^15^N chemical shift scale (39.3 ppm; Bertani et al., 2014).

### Deuterium Order Parameters

The deuterium order parameters (S_CD_) of each CD_2_ and CD_3_ group was determined according to: ${\text{S}}_{\text{CD}}^{\text{i}}{=\frac{4}{3}\frac{\text{h}}{{\text{e}}^{2}\text{qQ}}\Delta }^{\text{i}}\upsilon$, where Δ^i^ν is the quadrupolar splitting of segment i and (e^2^qQ/h) is the static quadrupole coupling constant (167 kHz) observed for deuterons within C–D bonds (Batchelder et al., 1983).

### Orientational Restraints From Oriented Solid-State NMR Spectra

A coordinate system was defined where the tilt angle is defined as the angle between the helix long axis and the membrane normal. The α-helical conformation had Ramachandran angles of (φ = −65°, ψ = −45°). The tilt and pitch angles were changed in 50 × 50 steps where at each orientation the corresponding ^15^N chemical shift and quadrupolar splitting were calculated (Michalek et al., 2013). The ^15^N chemical shift main tensor elements used for the calculations were 56, 81, and 223 ppm (Salnikov et al., 2009a). The orientation within the molecular frame of δ_22_ (81 ppm) is perpendicular to the peptide plane, the other two main tensor elements are situated in the plane of the peptide. The angle between the least shielded axis of the CSA tensor (223 ppm) and the N–H vector is 16° (Salnikov et al., 2009a).

The standard deviation of a Gaussian line shape takes into account orientational heterogeneity and several models were tested. In the first case the peptide was assumed static. Thereafter, independent wobbling (12° Gaussian distribution) and azimuthal fluctuations around the helix long axis (SD 40° or 80°) were taken into consideration by averaging the resonance values on the ensemble of orientations with the corresponding Gaussian distributions, similar to the dynamics of transmembrane sequences which have been tested previously including the WALP23 designer peptide (Bertelsen et al., 2009; Holt et al., 2010).

## Results

The DQB1 hydrophobic domain is characterized by a high degree of helical conformation when investigated in membrane environments (King and Dixon, 2010; Aisenbrey et al., 2019c). For NMR structural investigations the sequence was prepared by solid-phase peptide synthesis, including a ^15^N label at the amide bond position of leucine-15, i.e., in the central part of the polypeptide sequence, which has been found previously to be part of a well-structured region (Salnikov et al., 2019). This polypeptide was reconstituted into oriented bilayers of variable composition and investigated by proton-decoupled ^15^N solid-state NMR spectroscopy (Figure 1 and Figure S1).

When [^15^N-L15]-DQB1 is reconstituted into pure POPC membranes at a peptide-to-lipid ratio of 1 mol% the ^15^N solid-state NMR spectra are characterized by a major intensity at 196 ppm with an apparent line width at half-height (LWHH) of 4.5 ppm which superimposes on a broader resonance exhibiting a maximum at 179 ppm (Figure 1A). Qualitatively, this chemical shift range is indicative of transmembrane helical alignments (Bechinger and Sizun, 2003). When the peptide concentration is increased to 2 mol% a shoulder appears at 216 ppm while the previous intensities at 1% merge in to a broad line by a distribution of alignments in slow exchange (maximum 187 ± 13 ppm; Figure 1B).

In supported lipid bilayers made of POPC/SM-C18 95/5 mol/mol DQB1 exhibits the same maxima at 180 ppm and 196 ppm as well as a shoulder extending to 220 ppm (Figures 1C,D). The 2 mole% sample in this lipid mixture is dominated by the broad intensity that arises from an orientational distribution where only a small portion of the well-oriented mobile resonance remains (Figure 1D). Whereas, in the absence of SM-C18 and at 1 mol% peptide the 196 ppm resonance is roughly twice as intense when compared to that at 179 ppm (Figure 1A), the maxima are of about equal intensity in the presence of the sphingolipid (Figure 1C). At 4 mole% DQB1 the spectrum is similar to that recorded at 2 mole% (not shown).

A complementary DQB1 sequence was prepared were the Val14 site was labeled with ^15^N. This peptide was also reconstituted into supported POPC/SM-C18 95/5 mole/mole bilayers and investigated by ^15^N solid-state NMR spectroscopy (Figure 2A). The ^15^N chemical shift of 213 ± 4 ppm is characteristic of transmembrane helical sequences where the ^1^H-^15^N vector is close to parallel to the membrane normal.

When investigated in DMPC membranes the spectrum of 1 mol% [^15^N-L15]-DQB1 exhibits a single sharp line at 191.5 ± 4 ppm and a somewhat broader line at 189.5 ± 6.5 ppm when the concentration is doubled (Figures 1E,F). Interestingly, the same peptide was previously investigated in bicelles made from DMPC/DHPC (*q* = 3.2) which oriented with the membrane normal perpendicular to the magnetic field (i.e., 90° tilted from the spectra shown in Figure 1). The resulting ^15^N solid-state NMR spectra was characterized by a single sharp resonance at 90 ± 5 ppm, which agrees with the 189 ppm value observed for the supported lipid bilayer value observed at a sample alignment where the bilayer normal and the magnetic field coincide (Figure 1E). In DMPC membranes the ^15^N chemical shift of V14 is 208 ± 3.5 ppm (Figure 2B), corresponding to a value of 85 ± 7 ppm in DMPC bicelles, which are flipped by 90° relative to the glass plate samples (Figure 2C).

**Figure 3:**
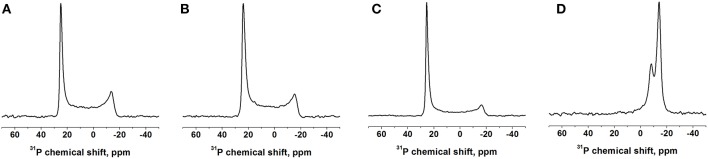
Proton-decoupled ^31^P solid-state NMR spectra of DQB1 samples made of **(A)** POPC, **(B)** POPC/SM-C18 at 293K and **(C)** DMPC at 310K, all oriented on glass plates with the membrane normal parallel B_o_. **(D)** DQB1 in DMPC/DHPC bicelles (q = 3.2, 28% w/v) which spontaneously align with the bilayer normal perpendicular to the magnetic field B_o_ of 7 Tesla. The peptide-to-lipid ratio is 1 mole% **(A–C)** or 2 mole% **(D)**.

The ^31^P NMR spectra of the mechanically oriented samples are all characterized by a predominant intensity around 25 ppm, indicative of oriented phosphatidylcholine membranes in their liquid crystalline state (Figures 3A–C and Figure S2). Some intensities extend up to -15 ppm showing some conformational and/or orientational heterogeneity at the level of the phospholipid head group (Bechinger and Salnikov, 2012). The bicellar sample exhibits two intensities at −14 and −8 ppm from DMPC and DHPC, respectively, indicative of a good magnetic alignment of the bicelles. In bicelles the first lipid predominantly constitutes the bilayer, the second is preferentially located in the rim of the bicelle, where different motional regimes explain the differences in chemical shift (Marcotte and Auger, 2005).

**Figure 4:**
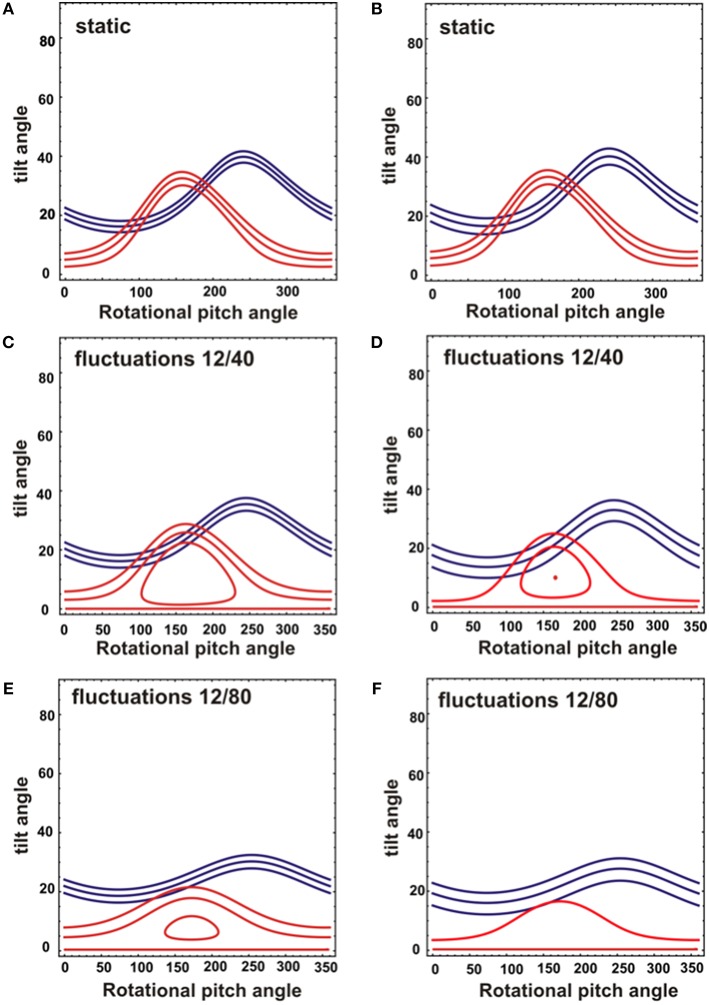
Analysis of NMR topological restraints for a DQB1 ideal helix reconstituted into mechanically oriented DMPC **(A,C,E)** or POPC/SM (95/5) membranes at 1 mole% **(B,D,F)**. The topological restraints from each NMR measurement are shown for ^15^N-V14 in red (208 ± 3.5 ppm for DMPC and 213 ± 4 ppm for POPC/SM), and for ^15^N-L15 in blue (191.5 ± 4 ppm for DMPC and 196 ± 6 ppm for POPC/SM). The restraints were obtained assuming either a static peptide alignment **(A,B)** or rocking and wobbling motions of the helix resulting in a Gaussian distribution of SD 12° and 40°, respectively **(C,D)** or of 12° and 80°, respectively **(E,F)**. The central line represents the main intensity, two additional restrictions were calculated from the ^15^N chemical shifts defining the LWHH to take into account orientational distributions and potential errors.

While the ^15^N chemical shift is a direct indicator of the approximate tilt angle of a helix (Bechinger and Sizun, 2003) the alignment of a helical domain relative the membrane surface can be determined more accurately when additional orientation-dependent NMR parameters are available (Bechinger et al., 2011; Gopinath et al., 2015; Ravula et al., 2017). The restriction analysis shown in Figure 4 shows the tilt/angular pitch angular pairs that agree with the ^15^N chemical shifts observed for [^15^N-L15]- and [^15^N-V14]-DQB1 in POPC/SM-C18 95/5 mol/mol. The restriction analysis was performed with a static peptide (Figures 4A,B) as well as peptides undergoing wobbling and rocking motions (Figures 4C–F). Comparing the different cases also provides insight into the precision of the absolute tilt/pitch angle analyses when based on a limited data set of two ^15^N chemical shifts (Michalek et al., 2013). Notably, the peptide alignment has to agree with the chemical shifts of both Val14 and Leu15, therefore, only angles where the red and blue traces intersect in the restriction plot have to be considered (Figure 4). For POPC/SM-18 95/5 mole/mole this is the case e.g. for a static peptide at tilt/pitch angular pairs around 17°/90° and 31°/185° (Figure 4B) or for a peptide exhibiting rocking motions around its long axis at a tilt angle of 20° (Figure 4D), or some intermediate situation.

The resonance at 180 ppm and the shoulder extending to 220 ppm appear and grow in parallel suggesting that they represent a single population of polypeptide that exhibits a range of alignments relative to the magnetic field direction, in a related manner to previous observations with residue A713 of the amyloid precursor protein (Itkin et al., 2017). We therefore simulated the spectral line shapes of a transmembrane helical domain that adopts a range of alignments represented by a cone-like distribution.

**Figure 5:**
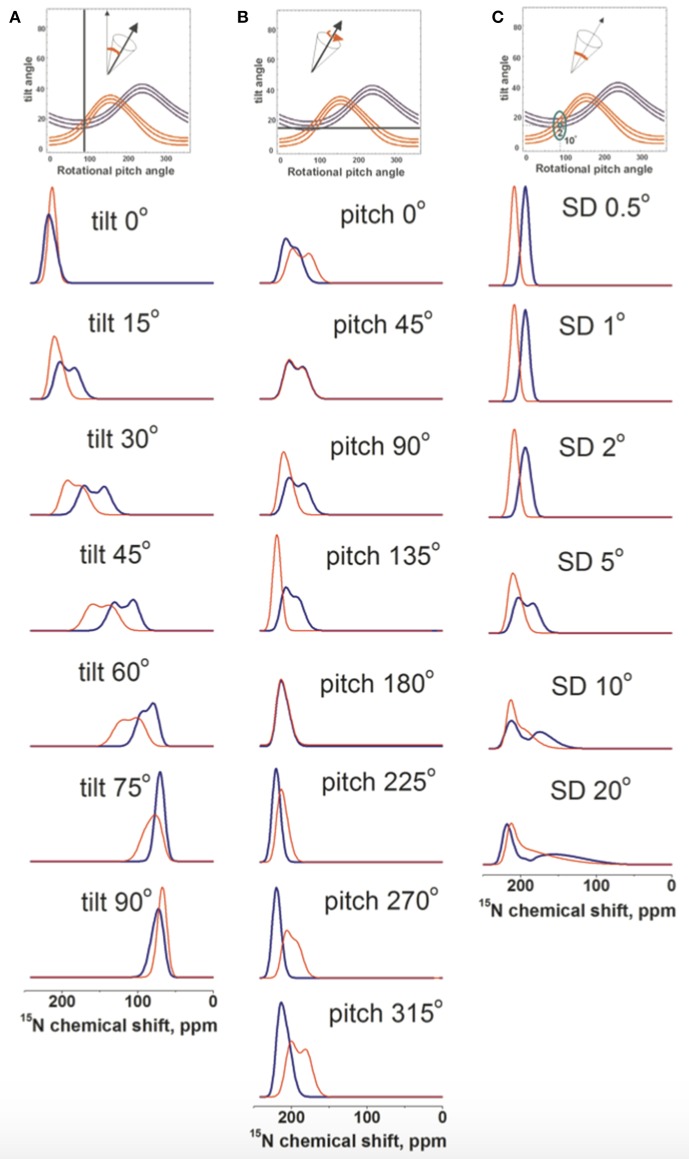
Spectral line shape simulations of ^15^N solid state NMR spectra of a peptide helix oriented at a tilt of 15°, a pitch of 90° and a Gaussian distribution of SD 5° i.e., an orientation of the DQB1 monomer in POPC/SM membrane found in Figure 4B. Whereas, two of these parameters are left constant one is systematically changed. **(A)** systematic change of the tilt angle, **(B)** of the pitch angle and **(C)** the Gaussian distribution. The simulated V14 resonance is in red, that of L15 in blue.

An initial alignment of tilt/pitch 15°/90° with for both angles a Gaussian distribution of SD 5° was chosen as a reference because it results from the restriction analysis shown in Figure 4B. In a first series of experiments the tilt angle was systematically varied while the pitch was kept constant. The resulting spectral line shapes of L15 and V14 were calculated under conditions where multiple alignments are in slow exchange and are represented by a Gaussian distribution with SD of 5°. As expected with increasing tilt angle the chemical shifts change from 220 to 65 ppm (Figure 5A). At the small tilt angles these simulations reproduce the broadening of the L15 line while the V14 resonance remains relatively sharp (Figure 5A) similar to the experimental observations (Figures 1A–D, 2A).

In another series of simulations the pitch angle was varied, i.e., the peptide was rotated around the helix long axis at a tilt of 15° and a Gaussian distribution of 5°. Whereas, the ^15^N chemical shift remained in the 200 ppm range the Gaussian distribution of alignments around the indicated values results in considerable broadening of the lines, which affect the Leu15 and Val14 resonances in a differential manner (Figure 5B). Finally, the helix was fixed at an average orientation of tilt/pitch 15°/90° but the Gaussian distribution was varied. Also in this case the main intensity is in the 200 ppm range but at SD ≥ 10° the line can extend far into the 100 ppm range (Figure 5C).

**Figure 6:**
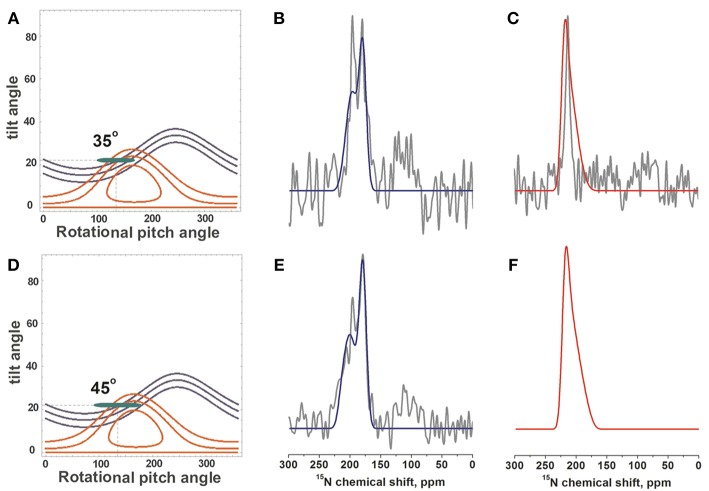
Simulations of ^15^N-DQB1 solid-state NMR spectra. When the DQB1 helix exhibits a tilt/pitch angular pair of 22°/135° with a range of alignments in slow exchange spectral line shapes and chemical shifts are observed which approximate the experimental spectra of Figures 1C,D, 2A (shown in gray). The Gaussian distribution of alignments exhibits a SD of 1° for the tilt and 35° for the pitch to reproduce the experimental spectra of 1 mole% DQB1 **(A–C)** or 45° for the experimental spectra of 2 mole% DQB1 **(D–F)**, respectively. **(B,E)** compare to the experimental spectra of ^15^N-L15, **(C)** to that of ^15^N-V14. The alignments that entered the simulation are shown in the contour plots **(A,D)** by the green ellipses.

Although some of the systematic simulations shown in Figure 5 represent the chemical shift average and the width of distribution rather well, including differences between V14 and L15, they fail to reproduce the detailed line shape of the broad component observed in Figures 1A–D, in particular the much higher intensity at 180 ppm when compared to the shoulder at 220 ppm. Therefore, a large number of simulations was set-up to find conditions where motions of the monomeric state result in a narrow line around 196 ppm for L15, but a broadened line shape when these motions are frozen (Figures 4B,D,F). A wide variety of peptide orientations and Gaussian distributions were tested until conditions reproducing well the experimental spectra could be identified. These are shown in Figure 6 and compared to the experimental spectra from Figures 1C,D, 2A. The simulations shown represent a tilt/pitch angular pair of 22°/135° where the tilt angle is well-defined but considerable motions around the helix long axis persist (Gaussian distribution of SD 35–45^o^). Notably, under conditions of fast motions the broad lines collapse into a single chemical shift of 190 ppm for Leu15 and 212 ppm for V14. The time scales for such a transition are in the 100 μs range.

**Figure 7:**
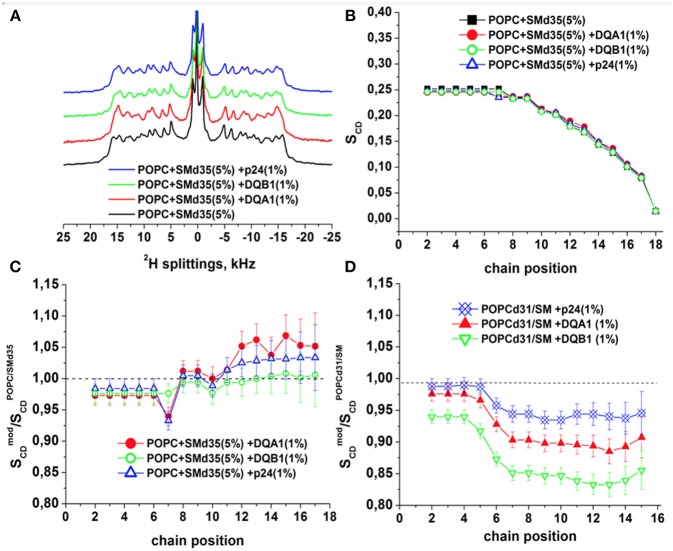
^2^H solid-state NMR spectra and order parameters of POPC/${\;}^{2}{\text{H}}_{35}$-SM (95/5 mole/mole) in the presence of 1 mol% p24, DQA1, or DQB1 transmembrane domain **(A-C)**. Relative order parameters of ${\;}^{2}{\text{H}}_{31}$-POPC/SM (95/5 mole/mole) in the presence of 1 mol% p24, DQA1, or DQB1 peptides are shown in **(D)** for comparison (original data from Aisenbrey et al., 2019a,c and are presented relative to the values obtained for ${\;}^{2}{\text{H}}_{31}$-POPC/SM-C18). The temperature was 310K.

To obtain further insight into the interactions of the transmembrane domains of p24, DQA1 and DQB1, the three polypeptide sequences were reconstituted into POPC/SM-C18 95/5 mol/mol membranes where either the palmitoyl chain of POPC or the stearoyl chain of SM-C18 were fully deuterated (i.e., ^2^H_31_-POPC or ^2^H_35_-SM-C18). Figure 7A shows the resulting deuterium solid-state NMR spectra of ^2^H_35_-SM-C18 in the pure lipid mixture with POPC as well as in the presence of 1 mole% of the three transmembrane helical peptide sequences. The spectrum is composed of the ^2^H quadrupolar splitting of individual CD_2_ segments where in analogy to other phospholipids the largest quadrupolar splitting is assigned to those segments closest to the head group, where the ^2^H NMR spectra of the first few segments overlap. The chances of cis/trans isomerization and of conformational dynamics increase along the fatty acyl chain thus the quadrupolar splittings decrease toward the membrane interior. The smallest quadrupolar splitting is associated with the terminal CD_3_ segment and an (almost) isotropic intensity is observed for residual HDO. When the spectra are deconvoluted individual splittings are obtained from which the segment specific order parameters are obtained (cf. section Materials and Methods). The order parameter as a function of segment number is shown in Figure 7B. Because the spectra of the most interfacial segments overlap a single value is obtained forming the “plateau region” (segments 2 to 4 in Figure 7B). Figures 7C,D shows the relative order parameter of ^2^H_35_-SM-C18 and ^2^H_31_-POPC, respectively, i.e., the ratio between the value in the presence of peptide and the pure lipid bilayer.

It should be noted that this assignment of quadrupolar splittings and chain position has early on be established using pure phosphatidylcholine membranes carrying deuterons at a single CD_2_ position (Seelig and Niederberger, 1974) and/or (Seelig and Seelig, 1974). To keep with previous publications we keep this assignment in Figures 7B–D. However, it cannot be excluded that in the presence of membrane-associated compounds local disturbances result in an exchange of the assignment order.

At 37°C the absolute order parameters of the plateau region of ^2^H_31_-POPC is 0.20 whereas ^2^H_35_-SM-C18 in the same lipid mixture exhibits a somewhat higher value of 0.25. Upon addition of the transmembrane sequences the order of the ^2^H_31_-POPC plateau region decreases by about 3% (DQA1, p24) to 7% (DQB1), while the one of ^2^H_35_-SM-C18 increases by 2–3% (Figures 7C,D). While some of these changes are hardly significant (<5%) more pronounced differences in the order parameter are observed in the hydrophobic region of the bilayer. Notably, for all the peptides the ^2^H_31_-POPC order decreases beyond segment 6 by 7% (in the presence of p24), 11% (DQA1) and 17% (DQB1), respectively (Aisenbrey et al., 2019c). In contrast, in the same mixture the order of the inner most segments of ^2^H_35_-SM-C18 remain constant (DQB1) or increase by 3-5% (DQA1, p24).

## Discussion

In order to better understand the protein-protein and protein-lipid interactions involved in regulatory processes the DQB1 transmembrane domain of the MHC II receptor was reconstituted into lipid bilayers and investigated by static oriented ^15^N solid-state NMR spectroscopy (Bechinger et al., 2011). When the sample is inserted into the NMR spectrometer with the membrane normal parallel to the magnetic field direction ^15^N chemical shifts around 200 ppm are indicative of transmembrane helical alignments (Bechinger and Sizun, 2003). Complementary information was obtained by investigating the fatty acyl chain order parameters of deuterated sphingomyelin, which has been shown to interact with a highly specific SM-C18 recognition motif also found in the transmembrane region of the receptor using ^2^H solid-state NMR spectroscopy.

### First Analysis and Assignment of Peaks

**Figure 8:**
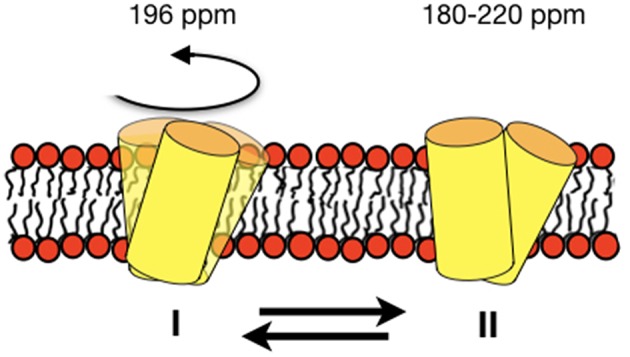
Schematic representation of an equilibrium between a fast-moving monomer and a less dynamic dimer of DQB1 is shown although the exact size of the complexes remains unknown from the experiments presented in this paper. The corresponding ^15^N chemical shifts are indicated on top of the membrane.

For DQB1 two populations with closely related alignment but different dynamics are observed in the ^15^N solid-state NMR spectra (Figures 1A–D). In POPC the 196 ppm resonance of DQB1 decreases relative to the 216 and 180 ppm intensities when the peptide-to-lipid ratio is increased, an observation which is suggestive that the signal at 196 ppm and at 180–220 ppm are from different oligomerization states, probably representing the monomeric and dimeric states observed in previous investigations (Figure 8) (King and Dixon, 2010; Dixon and Roy, 2019). The line broadening of the ^15^N spectra without much change in the average chemical shift suggests that oligomerization is accompanied by a change in dynamics but little change in the helical tilt angle (Figures 1, 6). Realignment has to be faster than 10^−3^−10^−4^ s for averaging of the 40 ppm chemical shift range to occur.

Interestingly, when investigated under the same conditions the spectrum of [^15^N-V14]-DQB1 is characterized by a single sharp resonance (Figure 2A and compare to Figure 1C) although it is a direct neighbor of L15. Because, the angle of the N-H vector between two consecutive sites in a helix is different by about 10°, anisotropic distributions of helix alignments or anisotropic motions can affect the labeled sites in a differential manner (Figures 5, 6). A concentration-dependent equilibrium of two populations was also observed for [^15^N-Leu16]-DQA1 (Aisenbrey et al., 2019c).

### Restriction Analysis and Line Shape Simulations

The restriction analysis shown in Figure 4 does not allow for a precise determination of the DQB1 helical topology. Nevertheless, the ^15^N chemical shifts clearly indicate a transmembrane alignment with a limited range of possibilities for the tilt/rotational pitch angles. A complete topological analysis has been obtained previously for other sequences where more angular restraints were available (Bechinger et al., 2011; Gopinath et al., 2015; Ravula et al., 2017; Aisenbrey et al., 2019a). This was achieved by preparing protein samples that were uniformly labeled with ^15^N and two-dimensional oriented solid-state NMR spectroscopy or by preparing additional peptides carrying different ^2^H and ^15^N labels. Both the restriction analysis (Figure 4) and the line shape simulations (Figure 6) are indicative that the DQB1 transmembrane domain adopts a tilt angle of about 20°. Whereas, at 1 mole% DQB1 the ^15^N solid-state NMR spectrum is characterized by a single or a motionally averaged pitch angle at higher peptide concentrations these motions are slowed down sufficiently to reveal a distribution of alignments characterized by a range of pitch angles (Figure 6). In contrast, DQA1 shows two distinct chemical shifts that suggest a significant change in topology upon homodimer formation (including a 10° change in tilt) (Aisenbrey et al., 2019c). The hydrophobic domains of DQB1 and DQA1 both encompass GxxxG motifs that have been identified to be involved in homo- and heterodimerization of these and other membrane anchor sequences (Travers et al., 1984; Russ and Engelman, 2000; King and Dixon, 2010; Dixon et al., 2014; Drake and Drake, 2016; Dixon and Roy, 2019). Notably, crossing angles of considerable size have previously been observed for other dimers interacting through the GxxxG motif (Smith et al., 2002; Sato et al., 2009; Itkin et al., 2017).

The assembly of DQA1/DQB1 heterodimers with the CD74 invariant chain is required for efficient ER export of the MHCII heterodimer and to prevent premature peptide binding (Dixon and Roy, 2019), much less attention has been given to the formation of homodimers (King and Dixon, 2010). One may hypothesize that the reversible formation of DQA1 and DQB1 homodimers is another regulatory element that is sensitive to changes in lipid composition and polypeptide concentration and provides a pool of building blocks that can be quickly made available for receptor assembly. Homodimer formation may even be a requirement to fine-tune the amount of monomeric DQA1 and DQB1 which would otherwise compete with heterodimer formation (King and Dixon, 2010). In addition, a function of homodimers apart from their role in antigen presentation cannot be excluded, and is also the case for CD74 for which a MHCII independent role in MIF signaling was reported (for a recent review see Jankauskas et al., 2019).

### Changes in Membrane Composition: Presence of SM and DMPC Membranes

The ^15^N solid-state NMR spectra of DQB1 show only subtle changes when 5 mole% SM-C18 is added to the POPC membranes (Figures 1A–D) as was also observed for DQA1 and for p24 (Aisenbrey et al., 2019a,c). This observation is suggestive that this lipid has only a small effect on the overall topology or on shifting the equilibria between different topological states. When reconstituted into DMPC membranes a single peak at about 190 ppm is observed which broadens when the peptide-to-lipid ratio is increased (Figures 1E,F). The ^15^N chemical shifts in DMPC are reduced by only about 5 ppm when compared to the sharp resonance observed in POPC or POPC/SM-C18 membranes (cf. Figures 1, 2) and therefore closely related membrane topologies are obtained in both membranes (Figure 4). However, the unique signal in DMPC is indicative that a single population predominates in the saturated membranes or that different populations and conformers are in fast exchange. Notably, also when investigated in magnetically oriented bicelles a single resonance at 85 ppm is observed for V14 (Figure 2C) and for L15 (Salnikov et al., 2019). Interestingly, the ^15^N solid-state NMR spectrum of [^15^N-G15]-DQA1 in bicelles exhibits a closely related chemical shift (Salnikov et al., 2019).

The differences in chemical shift distribution observed in bicelles (Figure 2C) (Salnikov et al., 2019) are reproduced in the mechanically oriented sample indicating that the lipid composition is their underlying cause. Therefore, the equilibria between the oligomeric states of DQB1 are most strongly affected by the lipid saturation, the peptide-to-lipid ratio and to some extent by the presence of SM-C18. Furthermore, in the case of [^15^N-L16]-DQA1 the presence of DQB1 had a relatively small effect (Aisenbrey et al., 2019c). It remains possible that the cytoplasmic and extracellular domains of the full-length protein also have an effect on monomer-dimer distribution and that the additional charged residues at the termini of the constructs investigated here shift this equilibrium.

### SM and POPC Order Parameters

Because SM-C18 has been shown to interact with the transmembrane domains of p24 as well as DQA1/DQB1 here we also investigated the effects of these three sequences with deuterated phospholipids using ^2^H solid-state NMR spectroscopy (Figure 7). Notably, although phosphatidylcholines and SM-C18 are thought to mix well in pure lipid membranes (de Almeida et al., 2003), in the presence of these transmembrane domains a very different behavior of ${\;}^{2}{\text{H}}_{31}$-POPC and ${\;}^{2}{\text{H}}_{35}$-SM-C18 has been observed (Figure 7). The already elevated order of ${\;}^{2}{\text{H}}_{35}$-SM-C18 further increases at segments ≥ 6 in the presence of peptides carrying the recognition sequence (DQA1 and p24) but not of DQB1 suggesting that the specific recognition of SM-C18 is important. At the same time the order of ${\;}^{2}{\text{H}}_{31}$-POPC decreases for segments located within the membrane interior. Notably, the changes in the ${\;}^{2}{\text{H}}_{31}$-POPC order in the presence of peptides are only observed in the mixed membranes with 5% SM, i.e., mixtures shown in Figure 7, but not in pure POPC lipid bilayers (Aisenbrey et al., 2019c). Whereas, the ${\;}^{2}{\text{H}}_{35}$-SM-C18 order increases most for DQA1 and p24, the ${\;}^{2}{\text{H}}_{31}$-POPC order decreases in the sequence DQB1 > DQA1 > p24. When DQA1/DQB1 is added in combination the changes were explained by the presence of DQB1 (Aisenbrey et al., 2019c).

At 1 mole% DQB1 in POPC/SM 95/5 mole/mole the Val14 and Leu15 ^15^N chemical shifts corresponds to tilt angles around 20° (Figure 4). Similar ^15^N chemical shifts and tilt angles have also been observed for the transmembrane domains of DQA1 and p24 (Aisenbrey et al., 2019a,c). Together with SM this arrangement has a disordering effect on the palmitoyl segments ≥ 6 of POPC. Interestingly, related POPC order parameter profiles were observed in the presence of e.g., PGLa and magainin 2, two cationic amphipathic peptides that reside in the membrane interface (Hallock et al., 2003; Kim et al., 2009; Salnikov et al., 2010; Grage et al., 2016; Harmouche and Bechinger, 2018; Aisenbrey et al., 2019b), suggesting that together SM-C18 and the transmembrane domains exhibit a positive curvature strain. Such an effect could facilitate budding during the p24-mediated formation of COP I vesicles (Dodonova et al., 2015) or be involved in the long-range interactions between various interactions partners of the MHC II complex (Dixon and Roy, 2019) as has been suggested for other sequences (Salnikov and Bechinger, 2011). The disordering of the POPC lipid packing is suggestive that the DQA1/DQB1 homo- and heterodimers adopt an hourglass shape in the presence of sphingomyelin. The exact nature of the molecular interaction between SM and the three TMDs remains unknown, but it is interesting to note that DQB1 has the highest disordering effect on POPC whereas for DQA1 and p24, which carry a SM-C18 recognition motif, the effect is attenuated. At the same time the SM stearoyl chain exhibits an ordering only in the presence of the motif suggesting that a specific association exists which orders the SM-C18 chain while decreasing some of the positive curvature strain sensed by POPC.

## Conclusions

In conclusion, the DQB1 solid-state NMR spectra are suggestive of an equilibrium of the transmembrane anchor sequences with closely related topology but different motional regime. The concentration-dependent spectral intensities suggest an about 20° tilted monomer that associates into dimers or higher order homo-oligomers (Figure 8). In POPC/SM-C18 membranes the SM-C18 order increases in the presence of p24 and DQA1 both carrying the SM-C18 lipid recognition motif when at the same time all three transmembrane domains strongly disorder the palmitoyl chain of POPC, DQB1 having the strongest effect. This latter observation suggests that together with sphingomyelin the transmembrane sequences arrange themselves in a manner to exhibit positive curvature strain an effect that is attenuated by the specific recognition motif. For the first time the ^2^H solid-state NMR investigations of deuterated SM-C18 has thereby provided valuable hints how the recognition motif affects the lipid structure and thereby also influences the protein-protein interactions of the transmembrane domains.

## Data Availability

The datasets generated for this study are available on request to the corresponding author.

## Author Contributions

ES, CA, and BP performed experiments. BBr helped in designing the experiments and the paper, and supervised lipid synthesis. BBe designed biophysical approach and wrote the paper.

### Conflict of Interest Statement

The authors declare that the research was conducted in the absence of any commercial or financial relationships that could be construed as a potential conflict of interest.

## Abbreviations

COP: Coat Protein
DHPC: 1,2-di-hexanoyl-*sn*-glycero-3-phosphocholine
DMPC: 1,2-di-myristoyl-sn-glycero-3-phosphocholine
DQA1-TMD: KK TETVV CALGL SVGLV GIVVG TVFII RGLRS KK
DQB1-TMD: KK QSKML SGIGG FVLGL IFLGL GLIIH HRSQK K
EEDQ: 2-ethoxy-1-ethoxycarbonyl-1,2-dihydroquinoline
EtOH: Ethanol
HFIP: Hexafluoroisopropanol
HPLC: High Performance Liquid Chromatography
LWHH: Line Width at Half Height
MALDI: Matrix-Assisted Laser Desorption Ionization
MeOH: Methanol
MHC: Major Histocompatibility Complex
MS: Mass Spectrometry
NMR: Nuclear Magnetic Resonance
p24-TMD: KK TNSRV VLWSF FEALV LVAMT LGQIY YLKR-CONH_2_
POPC: 1-palmitoyl-2-oleoyl-*sn*-glycero-3-phosphocholine
SD: Standard Deviation
SM: Sphingomyelin
SM-C18: N-Octadecanoyl-D-erythro-Sphingosylphosphorylcholine
TMD: Transmembrane Domain
TOF: Time Of Flight.
