# Supplementary material for: Structure, Topology, and Dynamics of Membrane-Inserted Polypeptides and Lipids by Solid-State NMR Spectroscopy: Investigations of the Transmembrane Domains of the DQ Beta-1 Subunit of the MHC II Receptor and of the COP I Protein p24
Source: Front Mol Biosci. 2019 Sep 24;6:83. doi: 10.3389/fmolb.2019.00083 (PMC6769064; doi:10.3389/fmolb.2019.00083)
Supplement: Supplementary file 1 [file Data_Sheet_1.pdf]

SUPPORTING INFORMATION

Structure, topology and dynamics of membrane-inserted polypeptides and lipids by solid-state NMR spectroscopy:

Investigations of the transmembrane domains of the DQ beta-1 subunit of the MHC II receptor and of the COP I protein p24

Evgeniy S. Salnikov[1], Christopher Aisenbrey[1], Bianca Pokrandt[2], Britta Brügger[2*], and Burkhard Bechinger[1,*]

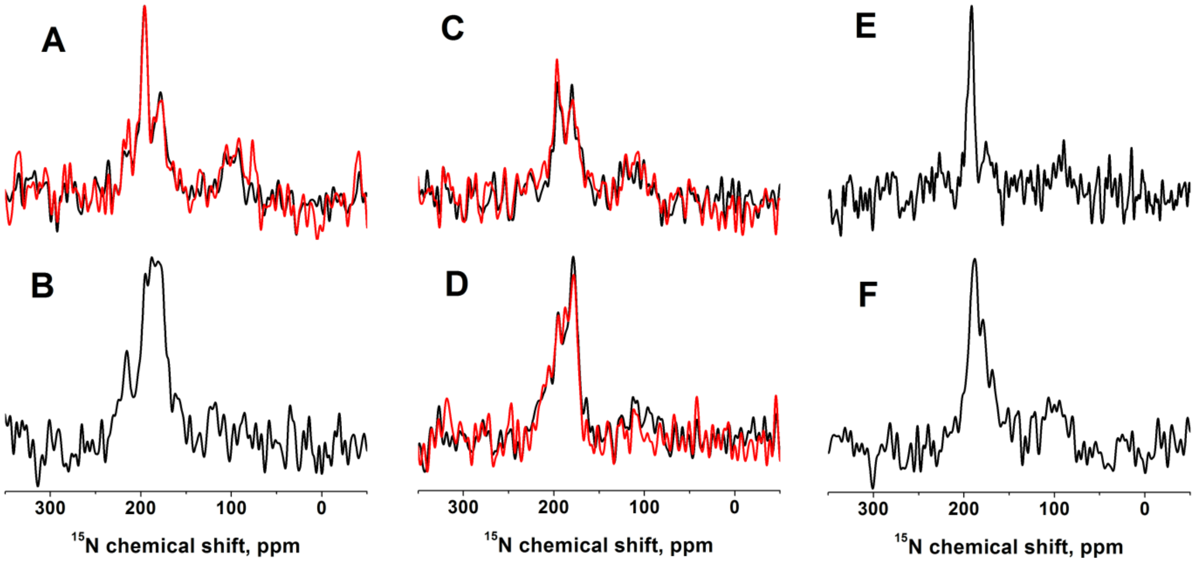

Figure S1: Some of the spectra shown in Figure 1 were re-run to test reproducibility of the line shapes. These are shown in red in panels A, C and D. The spectra show proton-decoupled cross-polarization ^{15}N solid-state NMR spectra of [^{15}N-Leu15]-DQB1 in mechanically oriented POPC (A,B), POPC/SM-C18 95/5 mole/mole (C,D) or DMPC (E,F) at 1 mole% (A,C,E) or 2 mole% (B,D,F). The red spectrum shown in C was previously published in (Aisenbrey et al., 2019).

In some spectra intensities < 100 are also observed for DQB1 (Fig. S1) which can be from in-plane oriented helices and/or non-oriented sample contributions (Bechinger and Sizun, 2003). Much more intense signals in this range have been observed for DQA1 at low P/L, which in the context of a more extensive series of experiments was tentatively assigned to peptides associated with the membrane interface (Aisenbrey et al., 2019).

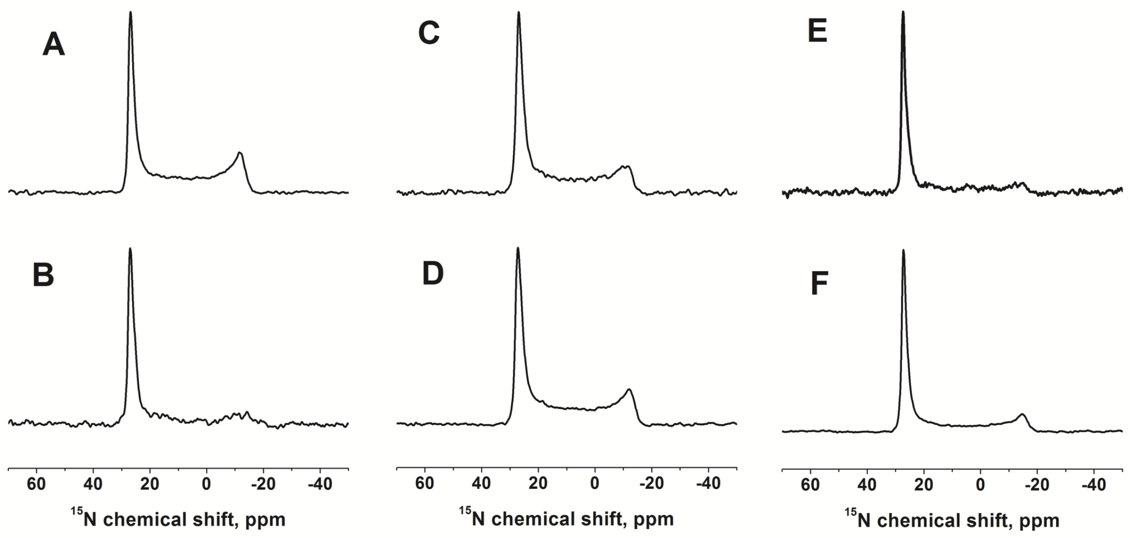

Figure S2. Proton-decoupled ^{31}P solid-state NMR spectra of DQB1 samples made of POPC (A,B), POPC/SM-C18 (C,D) at 293K and DMPC (E,F) at 310K, all oriented on glass plates with the membrane normal parallel B_o. DQB1 ^{15}N-labeled at position Leu15 was used in panels A (1 mole%), B (2 mole%), C (1 mole%) and E (2 mole%); or labeled at Val14 – in D (1 mole%) and F (1 mole%).

When the ^{31}P lines shapes are analyzed it becomes apparent that the intensities extending into the -15 ppm region are less pronounced for the 2 mole% samples (Fig. S2 B and E) when compared to the more dilute peptide-to-lipid ratios of 1% (Fig. S2A,C,D). This observation suggests that part of the non-oriented lipid is from the edges of the sample and contributes to the corresponding ^{31}P spectra. At the same time distortions of the lipid packing in the presence of peptide (which often is well-oriented) also results in different lipid bilayer alignments, contributions which increase with the thickness of the lipid layers (Hallock et al., 2003; Kim et al., 2009; Verly et al., 2009; Michalek et al., 2013). Third, charges at the membrane interface have been shown to alter the local conformation of the phospholipid head group and the corresponding chemical shift anisotropy but not the global alignment of the lipid (Scherer and Seelig, 1989). Because cross polarization is most efficient in the center of the coil the ^{15}N spectra represent the well-oriented part of the sample (cf. Fig. 2A where about 47% of the lipids appear to adopt a powder-like orientational distribution).

REFERENCES

Aisenbrey, C., Salnikov, E.S., and Bechinger, B. (2019). Solid-State NMR Investigations of the MHC II Transmembrane Domains: Topological Equilibria and Lipid Interactions. *J Membr Biol.* doi: 10.1007/s00232-019-00071-8. [Epub ahead of print]

Bechinger, B., and Sizun, C. (2003). Alignment and structural analysis of membrane polypeptides by 15N and 31P solid-state NMR spectroscopy *Concepts in Magnetic Resonance* 18A**,** 130 -145

Hallock, K.J., Lee, D.K., and Ramamoorthy, A. (2003). MSI-78, an Analogue of the Magainin Antimicrobial Peptides, Disrupts Lipid Bilayer Structure via Positive Curvature Strain. *Biophys J* 84**,** 3052-3060.

Kim, C., Spano, J., Park, E.K., and Wi, S. (2009). Evidence of pores and thinned lipid bilayers induced in oriented lipid membranes interacting with the antimicrobial peptides, magainin-2 and aurein-3.3. *Biochim. Biophys. Acta* 1788, 1482-1496.

Michalek, M., Salnikov, E.S., Werten, S., and Bechinger, B. (2013). Membrane interactions of the amphipathic amino-terminus of huntingtin. *Biochemistry* 52, 847-858.

Scherer, P.G., and Seelig, J. (1989). Electric Charge Effects on Phospholipid Headgroups. Phosphatidycholine in Mixtures with Cationic and Anionic Amphiphiles. *Biochemistry* 28, 7720-7727.

Verly, R.M., De Moraes, C.M., Resende, J.M., Aisenbrey, C., Bemquemer, M.P., Pilo-Veloso, D., Valente, A.P., Alemida, F.C., and Bechinger, B. (2009). Structure and membrane interactions of the antibiotic peptide dermadistinctin k by solution and oriented 15 N and 31 P solid-state NMR spectroscopy. *Biophys.J.* 96, 2194-2203.
